# TIGAR Promotes Tumorigenesis and Protects Tumor Cells From Oxidative and Metabolic Stresses in Gastric Cancer

**DOI:** 10.3389/fonc.2019.01258

**Published:** 2019-11-19

**Authors:** Zhenhua Liu, Yue Wu, Yingqiu Zhang, Menglang Yuan, Xuelu Li, Jiyue Gao, Shanni Zhang, Chengjuan Xing, Huamin Qin, Hongbo Zhao, Zuowei Zhao

**Affiliations:** ^1^Department of General Surgery, The Second Affiliated Hospital of Dalian Medical University, Dalian, China; ^2^Institute of Cancer Stem Cell, Dalian Medical University, Dalian, China; ^3^Department of Anesthesia, Dalian Maternal and Child Health Care Hospital, Dalian, China; ^4^Department of Pathology, The Second Affiliated Hospital of Dalian Medical University, Dalian, China; ^5^National Cancer Center/National Clinical Research Center for Cancer/Cancer Hospital and Shenzhen Hospital, Chinese Academy of Medical Sciences and Peking Union Medical College, Shenzhen, China

**Keywords:** apoptosis, cell cycle checkpoints, glycolysis, heterografts, stomach neoplasms

## Abstract

Cancer cells adopt glycolysis to facilitate the generation of biosynthetic substrates demanded by cell proliferation and growth, and to adapt to stress conditions such as excessive reactive oxygen species (ROS) accumulation. TIGAR (TP53-induced glycolysis and apoptosis regulator) is a fructose-2,6-bisphosphatase that is regulated by p53. TIGAR functions to inhibit glycolysis and promote antioxidative activities, which assists the generation of NADPH to maintain the levels of GSH and thus reduces intracellular ROS. However, the functions of TIGAR in gastric cancer (GC) remain unclear. TIGAR expression levels were detected by immunoblotting and immunohistochemistry in gastric cancer samples, along with four established cell lines of GC. The functions of TIGAR were determined by utilizing shRNA-mediated knockdown experiments. The NADPH/NADP^+^ ratio, ROS, mitochondrial ATP production, and phosphorus oxygen ratios were determined in TIGAR-depleted cells. Xenograft experiment was conducted with BALB/c nude mice. TIGAR was up-regulated compared with corresponding non-cancerous tissues in primary GCs. TIGAR knockdown significantly reduced cell proliferation and increased apoptosis. TIGAR protected cancer cells from oxidative stress-caused damages, but also glycolysis defects. TIGAR also increased the production of NADPH in gastric cancer cells. TIGAR knockdown led to increased ROS production, elevated mitochondrial ATP production, and phosphorus oxygen ratios. The prognosis of high TIGAR expression patients was significantly poorer than those with low TIGAR expression. Taken together, TIGAR exhibits oncogenic features in GC, which can be evaluated as a target for intervention in the treatment of GC.

## Introduction

Unlike normal-behaving cells, that depend on oxidative phosphorylation processes in the mitochondria to produce essential energy for cellular physiology, malignant cells shift their metabolism toward glycolysis, albeit under conditions with adequate oxygen, to facilitate the generation of biosynthetic substrates demanded by cell metabolism, and to cope with stress conditions like ROS [reactive oxygen species (ROS)] accumulation. These strategies employed by cancer cells are termed “the Warburg effect” (1). Glycolytic pathways help cancer cells to grow and to adapt to challenging microenvironments in the reprogramming of metabolic pathways have been closely implicated in tumorigenesis (2). A number of oncoproteins and tumor suppressors have been revealed to contribute to such metabolic reprogramming (3, 4). For instance, TIGAR, the TP53-induced glycolysis and apoptosis regulator, has been shown to plays an important role in cellular glycolysis and biosynthesis, thus acting as an oncogene (5–8).

TIGAR inhibits glycolysis and transfers metabolic intermediates to alternative pentose phosphate pathway, which functions to generate ribose−5-phosphate for nucleotide synthesis and to yield NADPH to maintain the levels of glutathione (GSH) and thus lower intracellular ROS (5, 9, 10). ROS can oxidize and damage mitochondrion in eukaryotic cells, which leads to mitochondrial dysfunction and cell injury. Therefore, TIGAR protects cells against damages by influencing glycolysis and regulating the intracellular GSH and ROS levels, leading to suppressions in both apoptosis and autophagy while contributing to cell growth and proliferation (9, 11).

The elevated levels of TIGAR have been shown for several human malignancies including breast cancers (12), nasopharyngeal carcinoma (13), colorectal cancer (14), and glioblastoma (15). The down-regulation of TIGAR has been correlated with cancer growth inhibition. However, the functions of TIGAR in GC so far remain unclear. In the present study, we reveal that TIGAR was highly expressed in primary GC samples, which protected tumor cells from oxidative and metabolic stresses, and TIGAR depletion led to efficient inhibition of tumor growth.

## Materials and Methods

### GC Cell Lines and Tissue Samples

Human gastric cancer cell lines SGC7901, AGS, MKN74, and BGC823 were obtained from the research center of Dalian Medical University and cultured with Roswell Park Memorial Institute 1640 media (RPMI1640, GIBCO, 11875119) supplemented with fetal bovine serum (10% FBS, WISTEN., 086150008) and antibiotics (penicillin and streptomycin). Cells were maintained at a humidified atmosphere in a CO_2_ incubator (37°C). Cells were passaged by trypsinization (0.25%) every 2 days.

A total of 32 GC samples were collected from surgical specimens of patients who were diagnosed and had not undergone chemotherapy before gastrectomy in the GI department, the Second Hospital of Dalian Medical University. Gastric cancers and corresponding adjacent tissues of all patients were cryopreserved in −80°C. The procedures have been approved by the Medical Ethics Committee of the Second Affiliated Hospital of Dalian Medical University. Signed informed consent forms were obtained from all participants.

### Western Blot Analysis

Proteins were extracted from cells, mouse tumor specimens, and frozen human gastric cancer specimens with RIPA lysis buffer as described previously (16). Protein concentration was determined with a G250 standard curve which was detected on an Enspire2300 spectrometer with OD 595 nm. Protein samples were fractionated by SDS-PAGE before transferred to nitrocellulose membranes. Samples were blocked with 4% (w/v) fat-free milk in PBS with 0.1% of Tween 20 (PBS/T), washed with PBS/T, and then incubated with primary antibodies for overnight at 4°C: TIGAR (1:2,000, Abcam, ab37910), Cyclin D1(1:1,000, SANTA CRUZ, F2513), BCL-2(1:1,000, CST, 2876. After PBS/T washes, Blots were treated with fluorescent secondary antibodies (1:15,000, Thermo, Goat anti-Mouse or Goat anti-Rabbit antibodies) for 1 h. Antibody labeling was detected using an Odyssey Infrared Scanner (LI-COR). The intensities for signal bands were analyzed by quantification using the Image Studio programme. β-actin or tubulin was probed as loading control (1:5,000, Abcam or Sigma).

### Stable Cell Lines

To inhibit TIGAR expression, the two short hairpin (shRNA) expressing vectors matching region 181 to 190 (TRC Number: TRCN0000051389 shRNA: 5′-CCGGGCTGCTGGTATATTTCTGAATCTCGAGATTCAGAAATATACCAGCAGCTTTTTG-3′, Sh-TIGAR B5), and region 605 to 614 (TRC Number: TRCN0000051390shRNA:5′-CCGGGACAGCGGTATTCCAGGATTACTCGAGTAATCCTGGAATACCGCTGTCTTTTTG-3′, Sh-TIGAR B6) were purchased from Sigma. Lentiviruses were prepared using transient transfection with Lipofectamine 3000 reagent (Invitrogen, 11668019) in HEK293T cells. To establish stable TIGAR knockdown cells, lentivirus-infected cells were treated with puromycin 24 h post infection. Cells were collected for Western blot to validate knockdown efficiency. The pCDH plasmid was a generous gift from Prof. Han Liu (Dalian Medical University) and used to establish TIGAR overexpression cell lines (17). Positive cell lines were selected through puromycin treatment and validated by Western blotting.

### Flow Cytometry

Cell apoptosis was measured through performing double staining using fluoresceinisothiocyanate (FITC)-conjugated Annexin V and propidium iodide (Kengene KGA108). Cell lines were treated with 50–100 uM of hydrogen peroxide (H_2_O_2_) for 6 h or with 5 mM of 2-deoxy-D-glucose (2-DG) (Sigma-Aldrich, USA) for 48 h to induce apoptosis. Cells were collected by trypsinization and washed with PBS before counted. Half million of cells per condition were analyzed with a flow cytometer (BD ACCURI C6). Raw data were analyzed using the Cell Quest Pro software (CFlow). Cell cycle distribution was measured using propidium iodide (PI, Biouniquer, BU-AP0103) staining. Cultured cells were harvested and fixed with ethyl alcohol (70%) at 4°C, before PI incubation and detection with the flow cytometer.

### Cell Viability

Tumor cell growth was assessed by 3-(4, 5-dimethylthiazol- 2-yl)-2, 5-diphenyltetrazolium bromide (MTT, Sigma, M5655) assays. Cells were seeded into 96-well plates (1,000 cells per well) and cultured in complete media for 12–48 h prior to MTT assays. Relative percentages of cell growth were calculated using OD measurements at 570 and 630 nm from a plate reader (PerkinElmer Enspire 2300). Quintuplicate wells were examined from each condition, and experiments were performed with three independent repeats.

The long-term tumor cell proliferation was examined by carrying out colony formation assays. Cells with stable TIGAR shRNA expression were seeded into 6-well plates (1,000 cells per well), with growth media refreshed every 3 days. After 2 weeks, colonies were Giemsa-stained for 15 min before PBS washes, and photographed on Biorad imager (ChemiDoc XRS+). Quantification was performed using Image Pro plus.

### Measurement of NADPH/NADP^+^ Ratio

NADP^+^ and NADPH were measured with the NADPH/NADP^+^ assay kit (abcam65349). Tissue and cell samples were extracted and stored immediately at −80°C. All samples are prepared in duplicate. NADPH standard, Developer and enzyme mix are prepared ahead of time. NADPH Standard Curve was also prepared. The samples were thawn on ice when they were ready to be tested. Then, processed following the manufacturer's instructions. NADP/NADPH Ratio is calculated as: *NADP*/*NADPH ratio* = (*NADPtotal*–*NADPH*)/*NADPH*.

### Mitochondrion Extraction

Mitochondria were extracted from gastric cancer cells (AGS and SGC-7901) using homogenization method. Cultured cells were collected (5 × 10^6^) and washed with PBS twice. Harvested cells were transferred to 15 ml tube and washed once with homogenization buffer (20 mM HEPES pH 7.5, 200 mM mannitol, 70 mM sucrose, 1 mM EDTA, 1 mM EGTA). Cells were then resuspended in 0.5 ml of homogenization buffer and passed through a 23G needle with force. Nuclear fraction was spun down by centrifugation at 600 × g for 10 min. Post-nuclear supernatant was pelleted at 7,000 × g at 4°C to precipitate mitochondria. Pellet was resuspended in homogenization buffer.

### Measurement of Mitochondrial Respiration

Oxygen consumption through mitochondria was examined with a dissolved oxygen electrode (Shanghai INESA Scientific Instrument Co., Ltd, Rex JPB-607A, China) in 1 mL respiratory buffer (5 mM, 220 mM mannitol, 20 mmpl/L MgCl_2_ KCl, 5 mM and 75 mM, Sucrose, 20 mM Tris-HCl, 0.1 mM EDTA, pH = 7.4) at 25°C. Stabilized oxygraph readings of O_2_ concentration for 1 min. Mitochondrial suspension (1 mg/ml) was added in respiratory buffer with mitochondrial complex-I substrate glutamate at 5 mM and malate at 5 mM or mitochondrial complex- II substrate succinate at 5 mM. After 2 min, 5 μL of ADP was added to start state III respiration. Different phases of mitochondrial respiration recorded were state I (only mitochondria), II (mitochondria + substrate), III (mitochondria + substrate + ADP), and IV respiration. State IV respiration was observed after completion of state III respiration. P/O = ADP (nM)/(III-IV) (18).

### ATP Production

ATP Production was measured by absorption photometric method at 750 nm. Isolated mitochondria and hexokinase (MB0049) were added into ATP synthesis buffer (0.25 m sucrose, 22 mM glucose, 5 mMM KH_2_PO_4_, 2 mM MgCl_2_, 2 mM ADP, 1 mM EDTA, 10 mM Tris, pH = 7.5), reacted on 30°C water bath for 10 min. 50 nM sodium succinate was added into reaction system on 30°C water bath for 10 min. Stopped with stopping buffer (30%TCA), centrifuged at 4,500 rpm for 5 min. 300 μL ammonium molybdate (2.5%) and 200 μL amino-naphthalsulfonic were added into supernatant dilution at 20°C for 10 min. KH_2_PO_4_ standard was used as Pi standard curve. ATP Production Rate = Pi(nM)/(mg.min) (19).

### Xenograft Mouse Model

SGC7901 and TIGAR stable knockdown SGC7901 cells (1 × 10^6^ per mouse) were subcutaneously inoculated into the oxters of 6-week-old female BALB/c nude mice (Beijing Vital River Laboratory Animal Technology Co., Ltd.). Ten days after tumor formation, the status of the mice and the sizes of the tumor were measured and recorded every day. The tumors were harvested on the 17th day, both the tumors and the mice were weighed and recorded. All procedures were approved and monitored by the local Animal Care and Use Committee at Dalian Medical University.

### Immunohistochemistry

In total, 47 resected gastric cancer samples with adjacent normal tissues were retrieved from the specimen collection from the GI department of the Second Affiliated Hospital of Dalian Medical University (Dalian, China). The procedures have been approved by the Medical Ethics Committee of the hospital. Signed informed consent forms were obtained from all patients participated. The samples have been formalin-fixed and paraffin-embedded, from which serial sections (4 μm) were prepared. Antigens were retrieved with the Bond Epitope Retrieval Solution using the Bond-max immunostainer (Leica Microsystems,). A polymer detection system was applied using the immunostainer with anti-TIGAR antibody (1:6,000 dilution, Abcam) as per manufacturer's instructions. The slides were inspected by two independent pathologists without knowing patient outcomes. In total, over 2,500 cells were counted for each sample, which was semi-quantitatively scored through calculating percentages of positive stained cells times the staining intensity (18). The intensity was scored as follows: 0, no dying, 1, light yellow, 2, brown, 3, dark brown. The IHC scores ranged from 0 to 400.

### Tissue Microarray

The tissue chip was produced by Shanghai Biochip Company, Ltd., Shanghai, China. All tissue samples were examined by routine pathological hematoxylin-eosin (HE) staining, with secondary diagnosis performed by experienced pathologists. Using a tissue chip production apparatus (Beecher Instruments, Inc), a gastric cancer tissue array with matched adjacent tissue containing 180 array block points (HStm-Ade180Sur-06) was completed, and finally 84 patients were enrolled for analysis. All patients involved have signed on the informed consent forms. This was approved by Medical Ethics of Taizhou Hospital, Zhejiang province.

### Statistical Analysis

Quantified data of Western blot were acquired through Image Studio (Licor). Differences in mean expression levels, cell viability, tumor volume, and tumor weight were analyzed by Student's *t*-test. The results were considered significant when a *P* value < 0.05 was obtained. Kaplan-Meier survival analysis and log-rank tests were employed to perform survival univariate analysis, match grade data were analyzed by Wilcoxon, *p* < 0.05 is considered as statistical significance. All statistical analyses were conducted with SPSS23.0.

## Results

### TIGAR Is Up-Regulated in GC

**Figure 1:**
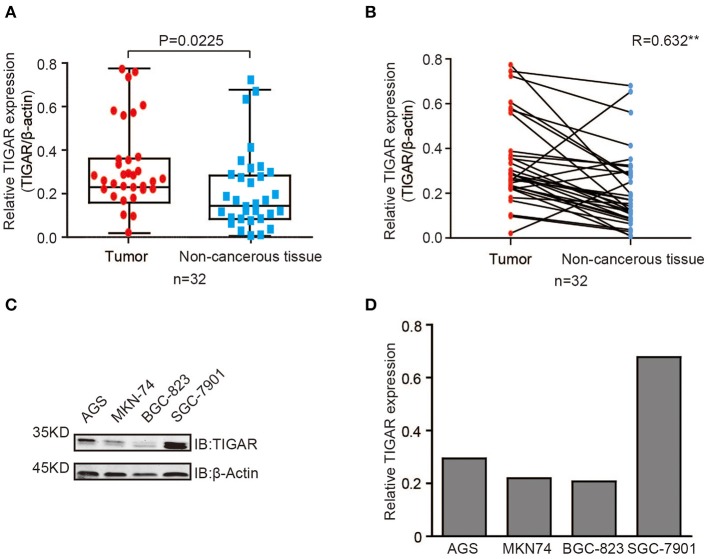
TIGAR is up-regulated in GC. **(A,B)** Analysis of TIGAR expression by Western blots using 32 primary GCs and paired non-cancerous tissues. Correlation of TIGAR expression between tumor and paired non-cancerous tissues was calculated by SPSS Statistics 23. In most GC tissues, TIGAR was up-regulated compared with paired non-cancerous tissues **(C,D)** TIGAR expression in four different GC cell lines (AGS, MKN74, BGC823, and SGC7901) by Western blot analyses and quantification data. TIGAR was expressed in all these four GC cell lines.

To detect the expression level of TIGAR in GCs, we first conducted Western blot using 32 primary GCs and corresponding paired non-cancerous tissues (Figure S1). In most GC tissues, TIGAR was up-regulated compared with paired non-cancerous tissues (Figures 1A,B). To further explore the function of TIGAR in GC cells, we conducted Western blot using four GC cell lines (AGS, MKN74, BGC823, and SGC7901). We found that TIGAR was expressed in all these four GC cell lines (Figures 1C,D). These results indicate that TIGAR may play an oncogenic role in GC tumorigenesis.

### TIGAR Is Causally Involved in the Tumor Progression of GC

**Figure 2:**
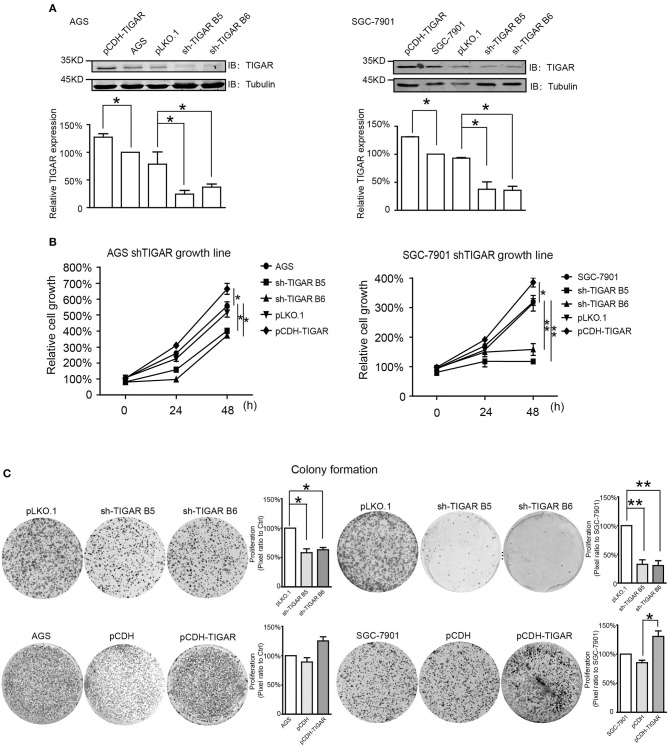
TIGAR is causally involved in the cell proliferation of GC. **(A)** Knockdown of TIGAR expression using two independent short hairpin RNAs (shTIGAR B5 and B6) and overexpression using pCDH construct in AGS and SGC7901 cells. The expression of TIGAR decreased to 35–37% and to 24–37% in AGS and SGC7901 cells with TIGAR knockdown, respectively. **(B)** MTT assays in both AGS and SGC7901 cells to investigate the short-term effects of TIGAR knockdown and overexpression on cell proliferation. TIGAR knockdown significantly reduced cell proliferation in AGS and SGC7901 cells, especially after 48 h culture, while the overexpression of TIGAR promoted cell proliferation. **(C)** Colony formation assays in both AGS and SGC7901 cells to investigate the long-term effect of TIGAR knockdown and overexpression. TIGAR knockdown significantly reduced the colony formation in AGS and SGC7901 cells, but cells stably overexpressing TIGAR showed increased growth. ^*^p < 0.05, ^**^p < 0.01.

Next, to explore whether TIGAR was causally involved in the tumor progression of GC, we knocked down TIGAR expression using two shRNAs (shTIGAR B5, shTIGAR B6) in two GC cell lines (AGS and SGC7901). The expression of TIGAR decreased to 35–37% and to 24–37% in AGS and SGC7901 cells with TIGAR knockdown, respectively, comparing with their normal control cells (Figure 2A).

**Figure 3:**
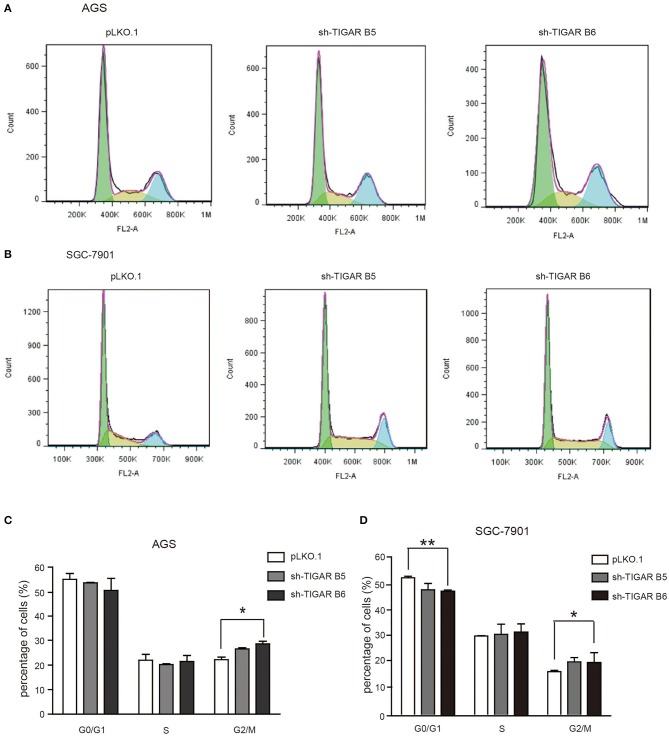
Knockdown of TIGAR modifies cell cycle progression. **(A,B)** Flow cytometry to analyze the alterations of cell cycle distribution by TIGAR knockdown. Cell cycle progression was modified after shRNA-mediated TIGAR knockdown **(C,D)** Quantification data of cells distributed at each stage of cell cycle. There is an increase in G2/M fractions. ^*^p < 0.05, ^**^p < 0.01.

To determine the short-term effects of TIGAR knockdown on cell viability, we employed MTT assay in both AGS and SGC7901 cells. TIGAR knockdown significantly reduced cell viability in AGS and SGC7901 cells, especially after 48 h culture, while the overexpression of TIGAR promoted cell viability (Figure 2B). In addition, this finding was validated by a long-term knockdown of TIGAR with colony-formation assay. TIGAR knockdown significantly reduced the colony formation in AGS and SGC7901 cells, but cells stably overexpressing TIGAR showed increased growth (Figure 2C). These data indicated that TIGAR was implicated in the tumor progression of GC. To explore the mechanism by which TIGAR regulates cell proliferation, flow cytometry was employed to assess the cell cycle distribution in SGC7901 and AGS cells. Cell cycle progression was modified after shRNA-mediated TIGAR knockdown, as shown by the increase in G2/M fractions (Figures 3A–D). Our data indicated that TIGAR knockdown was capable of significantly modifying cancer cell proliferation.

### TIGAR Protects Cancer Cells Against Oxidative Stress

**Figure 4:**
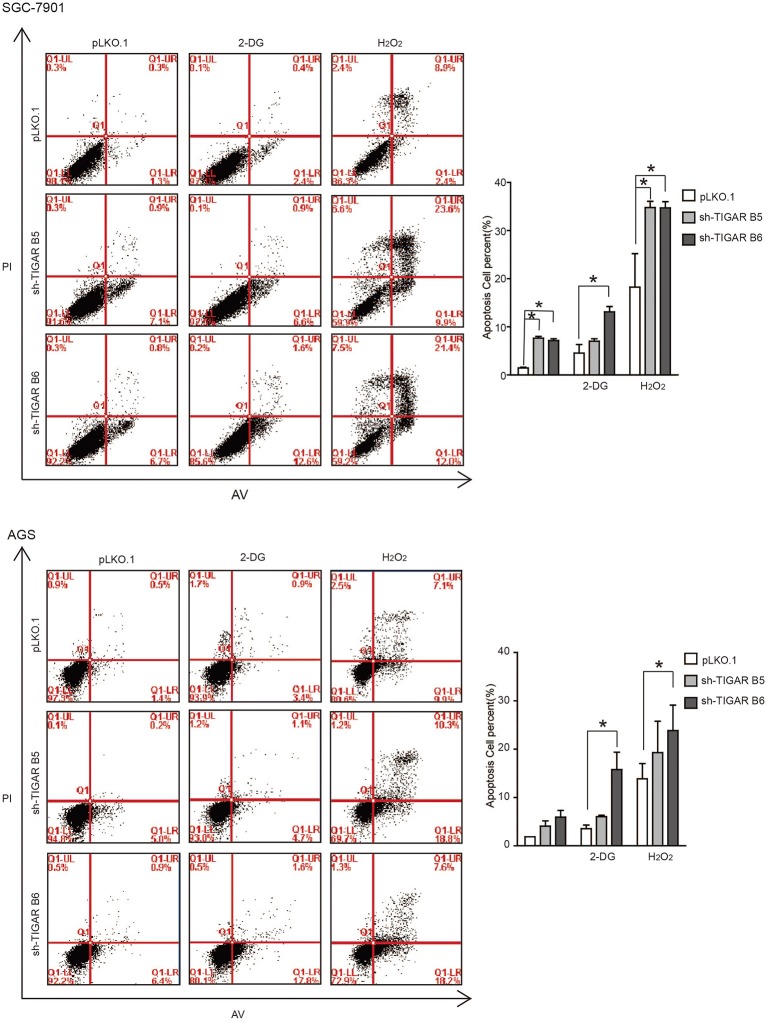
TIGAR protects cancer cells against oxidative stress and glycolysis inhibitor. Flow cytometry to analyze cell apoptosis in TIGAR knockdown SGC7901 and AGS cells treated with 50 μM H_2_O_2_ or 5 mM 2-DG. The percentages of apoptotic cells were increased after H_2_O_2_ treatment at the early and the total apoptosis quantiles (*P* < 0.05). The percentages of apoptotic cells were significantly increased in the early and total apoptosis quantiles in cells with TIGAR knockdown (*P* < 0.001). The combination of TIGAR knockdown with 2-DG addition significantly increased the cell apoptosis. ^*^p < 0.05.

It was reported that TIGAR protects cancer cells against ROS-induced cell apoptosis (14, 15, 19, 20). To determine the role of TIGAR in GC, we first induced an oxidative stress condition by adding hydrogen peroxide (H_2_O_2_). We observed that the percentages of apoptotic cells were increased after H_2_O_2_ treatment at the early and the total apoptosis quantiles (*P* < 0.05). Meanwhile, the percentages of apoptotic cells were significantly increased in the early and total apoptosis quantiles in cells with TIGAR knockdown (*P* < 0.001; Figure 4). These data indicated that TIGAR protected cancer cells from oxidative stress-induced cell deaths.

### TIGAR Knockdown Sensitizes GC Cells to Glycolysis Inhibitor

It is well-established that TIGAR slows down the glycolysis pathway. We thus hypothesized that TIGAR knockdown could facilitate the glycolysis process, and a combined treatment with glycolysis inhibitor (2-Deoxy-D-glucose: 2-DG) might result in an inhibition. 2-DG, a glucose analog, acts as a competitive inhibitor of glucose metabolism in preclinical and clinical applications (21, 22). Our results showed that the combination of TIGAR knockdown with 2-DG addition significantly increased the cell apoptosis (Figure 4). These data indicated that TIGAR knockdown sensitized gastric cancer cells to the glycolysis inhibitor.

### TIGAR Promotes the Production of NADPH and Reduces ROS

**Figure 5:**
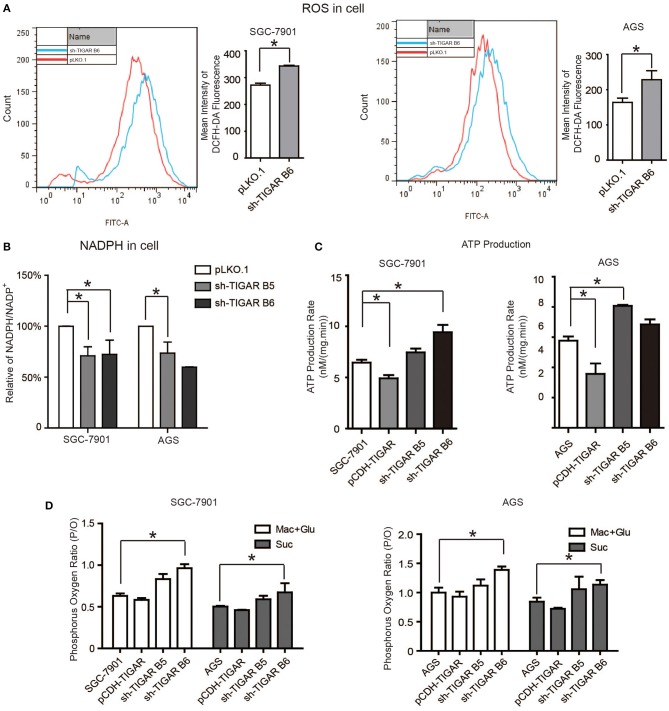
TIGAR regulates the production of NADPH and reduces ROS. **(A)** Flow cytometry assays to measure the levels of ROS in TIGAR knockdown and control cells. TIGAR knockdown significantly increased the ROS production. **(B)** NADPH /NADP^+^ assay to detect alterations of NADPH/NADP^+^ in TIGAR-depleted gastric cancer cells. TIGAR knockdown significantly reduced the generation of NADPH, leading to reduced NADPH/NADP^+^ ratios. **(C,D)** Mitochondria were isolated from control and TIGAR knockdown gastric cancer cells for ATP production and phosphorus oxygen ratio analyses. TIGAR expression levels were associated with ATP production and phosphorus oxygen ratios. ^*^p < 0.05.

It was reported that TIGAR inhibits glycolysis and generates NADPH to maintain the levels of GSH and thus down regulates intracellular ROS, which inhibits cell apoptosis and autophagy (9). Meanwhile, cancer cells can increase the NADPH levels to protect from the damages caused by ROS. Our data showed that TIGAR knockdown significantly increased the ROS production and reduced the generation of NADPH, leading to reduced NADPH/NADP^+^ ratios (Figures 5A,B). Further assays with mitochondria isolated from TIGAR knockdown cells showed that TIGAR expression levels were associated with ATP production and phosphorus oxygen ratios (Figures 5C,D). Taken together, these results suggest that TIGAR plays an oncogenic role via promoting the production of NADPH and regulating glycolysis.

### TIGAR Promotes Tumorigenesis in Nude Mice

**Figure 6:**
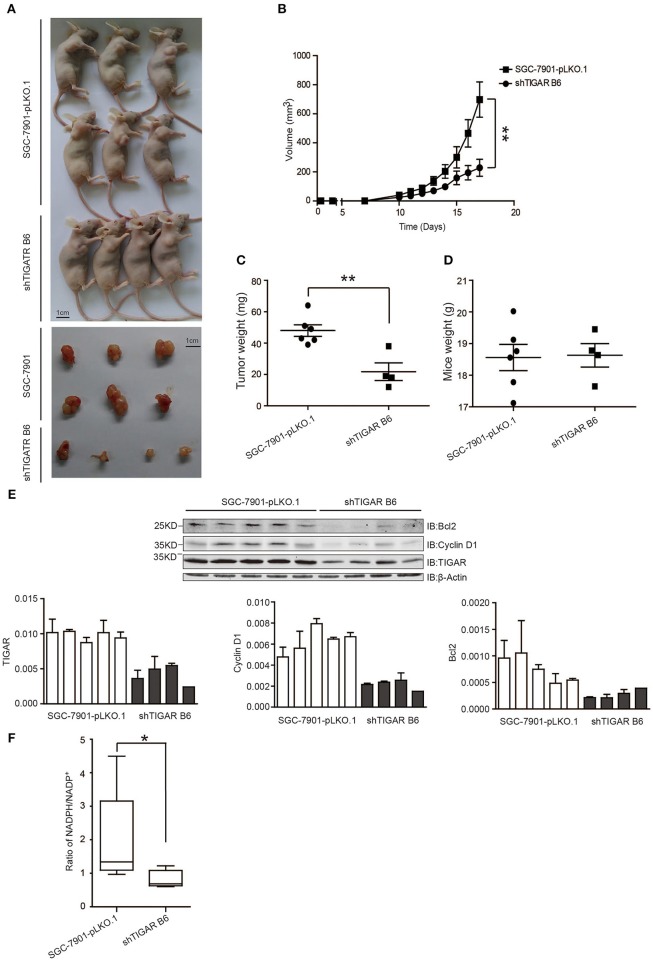
TIGAR promotes tumorigenesis in nude mice. **(A,B)** A xenograft nude mouse model to evaluate the effects of TIGAR *in vivo*. TIGAR knockdown tumors were smaller in size than the control tumors. **(C)** Comparison of tumor weight. TIGAR knockdown tumors had less weight compared to controls. **(D)** Comparison of mouse weight. There is no significant difference between the two groups. **(E)** Western blot to detect the expression of Bcl2, cyclin D1, and TIGAR. TIGAR knockdown significantly decreased the expression levels of cyclinD1 and bcl-2 *in vivo*. **(F)** NADPH /NADP^+^ assay to detect alterations of NADPH/NADP^+^ in xenograft tumors. TIGAR knockdown significantly reduced the NADPH/NADP^+^ ratios *in vivo*. ^*^p < 0.05, ^**^p < 0.01.

Finally, the *in vivo* effects of TIGAR were analyzed by transplanting SGC7901 cells with or without TIGAR knockdown into the oxters of nude mice. TIGAR knockdown tumors were smaller in size than the control tumors (Figures 6A,B) and had less weight compared to controls (Figure 6C). In terms of body weights of mice, there is no significant difference between the two groups (Figure 6D). Taken together, these data indicated that TIGAR played an oncogenic role in GC. Further, we detected the expressions of bcl-2, cyclinD1, and TIGAR in xenografts. Our data suggested that TIGAR facilitated cell proliferation by up-regulating cycinD1 and bcl-2 *in vivo* (Figure 6E). Also, in accordance with our *in vitro* results, the *in vivo* data showed that TIGAR knockdown significantly reduced the NADPH/NADP^+^ ratios (Figure 6F). Collectively, these data supported the notion that TIGAR played an oncogenic role in GC.

### Clinicopathological Relevance of TIGAR Expression

**Figure 7:**
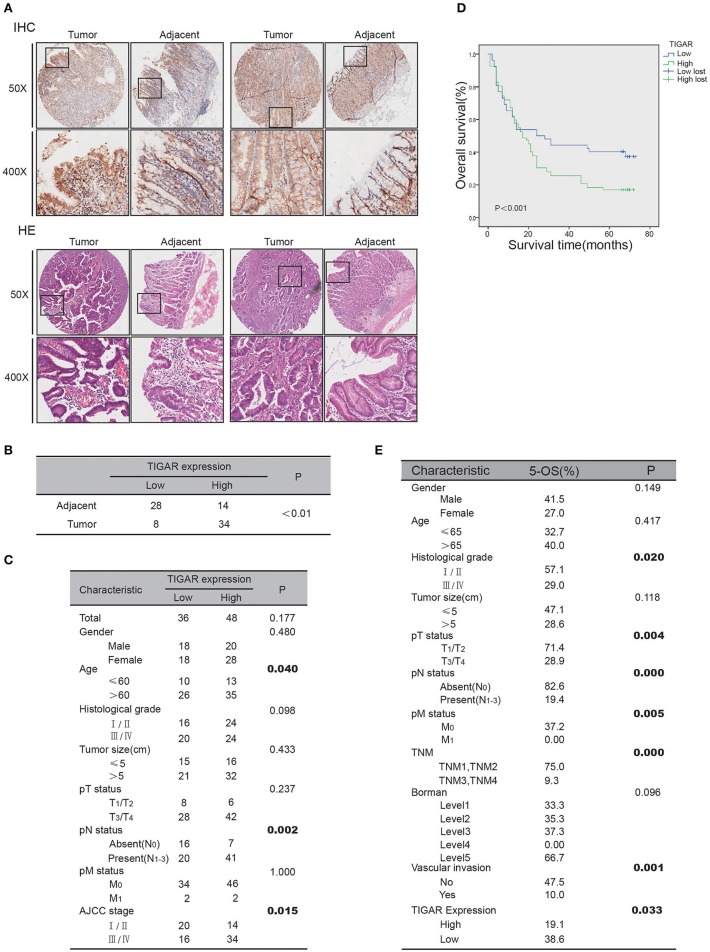
Clinicopathological relevance of TIGAR expression. **(A)** The IHC analysis of TIGAR protein expression from human gastric cancer tissue microarrays. **(B)** Comparison of TIGAR expression between tumor and adjacent tissues. The expression of TIGAR in gastric cancer tissues was significantly higher than that in adjacent non-cancerous tissues (*n* = 42, *p* < 0.01). **(C)** Correlation of TIGAR expression in relation to clinicopathologic variables of 84 gastric cancer patients. There was significant difference between TIGAR high expression and low expression patients in respect of mean age (*p* = 0.04), N staging (*p* = 0.002), AJCC staging (*p* = 0.015). **(D)** Overall survival of gastric cancer patients with high or low TIGAR expression was analyzed by Kaplan-Meier analysis. The 5-year survival rate of patients with high TIGAR expression was significantly lower than those with low TIGAR expression (*p* = 0.033). **(E)** The 5-year overall survival on different clinicopathological factors.

To assess the significance of TIGAR expression in gastric cancer, we carried out immunohistochemistry analysis using a gastric cancer tissue array. IHC results demonstrated that the expression of TIGAR in gastric cancer tissues was significantly higher than that in adjacent non-cancerous tissues (*p* < 0.01; Figures 7A,B). Furthermore, we examined the association of TIGAR expression with the clinicopathological parameters of patient tissues. As shown in Figure 7C, our results indicated that there was significant difference between TIGAR high expression and low expression patients in respect of mean age (*p* = 0.04), N staging (*p* = 0.002), AJCC staging (*p* = 0.015). High TIGAR expression was observed to be significantly correlated with elder age, lymph node metastasis, and advanced AJCC stages of gastric patients. We also carried out Kaplan-Meier survival analysis and log-rank test to assess the significance of TIGAR expression in the survival of gastric cancer patients. As shown in Figures 7D,E, the 5-year survival rate of patients with high TIGAR expression was significantly lower than those with low TIGAR expression (*p* = 0.033). Correlation of mRNA expression between TP53 (P53) and C12orf5 (TIGAR) was analyzed using sequencing data retrieved from TCGA (The Cancer Genome Atlas) STAD (stomach adenocarcinoma) dataset in UCSC Xena repository. As described in Figure S2, it appeared that correlation of TP53 and C12orf5 expression turned out to be more significant after filtering by age ( ≤ 60) and N (N0).

## Discussion

In the present study, we found that TIGAR was highly expressed in primary gastric cancers, which is consistent with previous findings of TIGAR expressions in several types of human cancers (12–15, 23). Although the effects of TIGAR have been reported previously in other type of cancer (13, 23–26), but still remained unexplored in gastric cancer. Therefore, it is important to examine the exact roles of TIGAR in gastric cancer to confirm its oncogenic implications, especially from the clinical perspective. The knockdown of TIGAR markedly reduced cell proliferation and increased cell apoptosis. TIGAR not only protected cancer cells from oxidative stress condition, but also inhibited the glycolysis process. TIGAR knockdown activated the glycolysis process, and a combined therapy with the glycolysis inhibitor resulted in a synergistic inhibition. Mechanically, TIGAR played an oncogenic role via the generation of NADPH. Furthermore, these findings were validated by *in vivo* experiments. The 5-year survival rate of patients with high TIGAR expression was significantly lower than those with low TIGAR expression.

Our data indicated that TIGAR knockdown modified cell cycle progression at the G2/M phase, and increased cell apoptosis. Madan et al. reported similar results that TIGAR knockdown released the G1 arrest and increased cell percentages at the G2/M phase in KB cells (27). It is well-known that, G0/G1 phase contains a check point prepared for DNA duplication, during which biomolecules such as nucleotides are synthesized. Similarly, G2/M phase contains another check point, when cell division is decided. At this phase, the following events, such as DNA injury, the amounts of substance for synthesis, and the volume of the cell, are detected, which functions to provide enough time for the repairment of DNA injury. It is well-known that there is common G0/G1 check point deficiency in cancer cells, while cancer cells will be selectively arrested at the G2/M phase when they encounter DNA injury, so G2/M phase is considered as an ideal target for cancer therapy. When cancer cells were arrested at this phase for a long time, the DNA injuries accumulate at the same time and induce cell apoptosis. Therefore, this offers us with an option for cancer therapy, through trying to arrest cancer cells at the G2/M phase.

Our findings revealed that TIGAR knockdown satisfied this demand, so TIGAR might serve as a promising target for GC therapy. To explore the potential mechanism, we analyzed the alterations of cell cycle markers cyclin D1. The results showed that cyclin D1 was decreased after TIGAR knockdown in *in vivo* experiment, but the detailed underlying mechanism needs further investigation.

In GC cells under oxidative stress, TIGAR knockdown significantly increased cell apoptosis compared with control cells, indicating that TIGAR played a protective role in cancer cell survival. It has been reported that TIGAR inhibits glycolysis and generates NADPH to maintain the level of GSH and thus lower intracellular ROS, which limits cell apoptosis and autophagy (10). Another study reported that the anticancer efficacy of epirubicin was enhanced after TIGAR was knocked down, owing to the elevation of intracellular ROS concentration and the increase of apoptosis, which could be partly blocked by the ectopic addition of NADPH and N-acetyl cysteine (NAC) (24). NADPH serves as the major reducing power against ROS in mammalian cells, which is also critical for the reductive biosynthesis of important biomolecules. Since NADPH is indispensable for rapid proliferating cells to survive, we therefore investigated the ratio of NADPH/NADP^+^ and found that TIGAR knockdown decreased the NADPH/NADP^+^ ratios from both *in vitro* and *in vivo* experiments, which is consistent with previous studies. Considering the established evidence that cancer cells need to maintain a suitable level of ROS to facilitate tumorigenesis, TIGAR plays an important role in intracellular ROS regulation through NADPH production. Depletion of TIGAR leads to ROS elevation, which likely serves as a main contributor to induced apoptosis in gastric cancer cell. Furthermore, to investigate the potential mechanism of the increased apoptosis after TIGAR knockdown, we analyzed the alterations of the anti-apoptosis marker bcl-2, and observed that bcl-2 was reduced after TIGAR knockdown. Hence, we drew a conclusion that TIGAR knockdown induced GC cell apoptosis through the reduction of NADPH and bcl-2, but other mechanically involved molecules in this process still need further investigations.

Metabolism is involved in virtually every aspects of cellular physiology. There is mounting evidence for cross-talks between signaling pathways and metabolic control in every multicellular organism studied. It is becoming clear that certain metabolic alterations are essential for malignant cancers. Essentially, the metabolic dependencies of cancer cells can be exploited for cancer treatment. Drugs targeting key metabolic control points that are important for aerobic glycolysis is worth of being investigated as potential cancer therapies. TIGAR functions as an inhibitor of glycolysis pathway which seems to be harmful for cancer cell survival. However, the knockdown of TIGAR induced an increase of cell apoptosis in GC cells. There might be a balance between TIGAR- reduced apoptosis and -inhibited glycolysis (23). In our study, silencing TIGAR or treatment with the glycolysis inhibitor (2-DG) alone induced limited cell apoptosis, while a combination of both caused increased cell apoptosis in GC cells. Therefore, TIGAR might become a potential therapeutic target for GC, and a combination with glycolysis inhibitors could be considered in future studies. To the best of our knowledge, the current study is the first report that revealed the function of TIGAR in gastric cancer cells.

In summary, our results revealed that TIGAR was highly expressed in GCs, consistent with results recently reported by Kim et al. (28). Patients who had high expression of TIGAR had poorer prognosis. In terms of function, TIGAR not only protects cancer cells from oxidative stress-caused damages, but also inhibits the glycolysis process. TIGAR plays an oncogenic role in GC tumorigenesis, and could possibly become a target for therapy for GC patients in the future.

## Data Availability Statement

All datasets generated for this study are included in the article/Supplementary Material.

## Ethics Statement

The studies involving human participants were reviewed and approved by Medical Ethics of the 2nd Affiliated Hospital of Dalian Medical University. The patients/participants provided their written informed consent to participate in this study. The animal study was reviewed and approved by Medical Ethics of the 2nd Affiliated Hospital of Dalian Medical University.

## Author Contributions

ZL, YW, and ZZ: conception and design, writing, review, and revision of the manuscript. ZL and YW: development of methodology. ZL, YW, YZ, MY, XL, JG, SZ, CX, HQ, and HZ: acquisition of data (acquired and managed patients, provided facilities, etc.). ZL, YW, YZ, MY, XL, SZ, CX, HQ, and ZZ: analysis and interpretation of data (e.g., statistical analysis, biostatistics, computational analysis). ZZ: study supervision.

### Conflict of Interest

The authors declare that the research was conducted in the absence of any commercial or financial relationships that could be construed as a potential conflict of interest.

## Supplementary Material

Figure S1 TIGAR expression in gastric cancer tissues. **(a)** TIGAR expression levels in gastric cancer tissues and corresponding non-cancerous tissues were examined by immunoblotting using paired samples as indicated. Actin blots show loading control. **(b)** Data from a were quantified by measuring TIGAR band intensities compared to corresponding actin band intensities.

Figure S2 Correlation of TP53 (P53) and C12orf5 (TIGAR) expressions. Correlation of mRNA expression between TP53 (P53) and C12orf5 (TIGAR) was analyzed using sequencing data retrieved from TCGA (The Cancer Genome Atlas) STAD (stomach adenocarcinoma) dataset in UCSC Xena repository. It appeared that correlation of TP53 and C12orf5 expression turned out to be more significant after filtering by age (≤ 60) and N (N0).
