# Supplementary figures and images for: TIGAR Promotes Tumorigenesis and Protects Tumor Cells From Oxidative and Metabolic Stresses in Gastric Cancer
Source: Front Oncol. 2019 Nov 19;9:1258. doi: 10.3389/fonc.2019.01258 (PMC6878961; doi:10.3389/fonc.2019.01258)

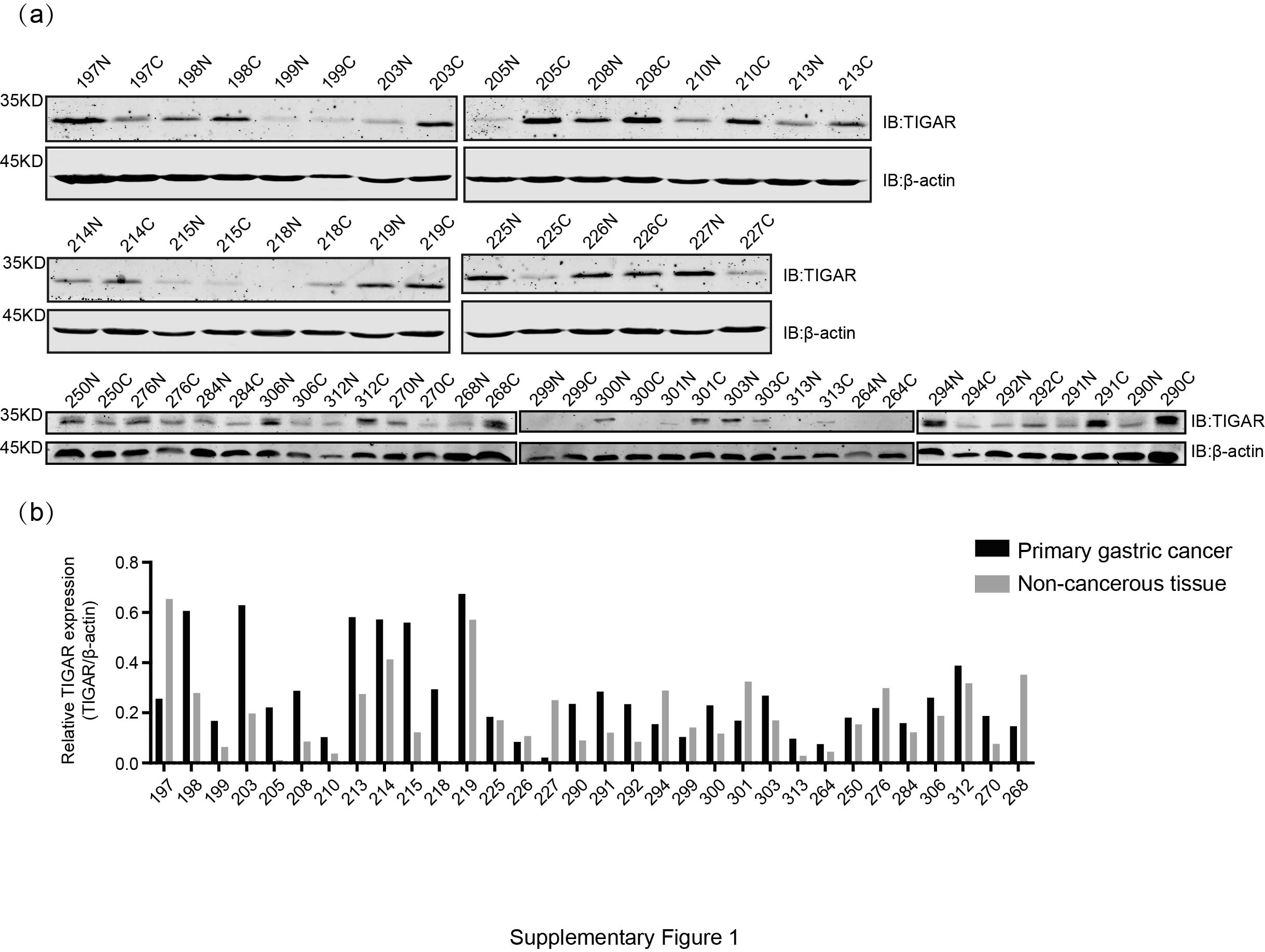

Supplement: Figure S1 — TIGAR expression in gastric cancer tissues. (a) TIGAR expression levels in gastric cancer tissues and corresponding non-cancerous tissues were examined by immunoblotting using paired samples as indicated. Actin blots show loading control. (b) Data from a were quantified by measuring TIGAR band intensities compared to corresponding actin band intensities. [file Image_1.JPEG]

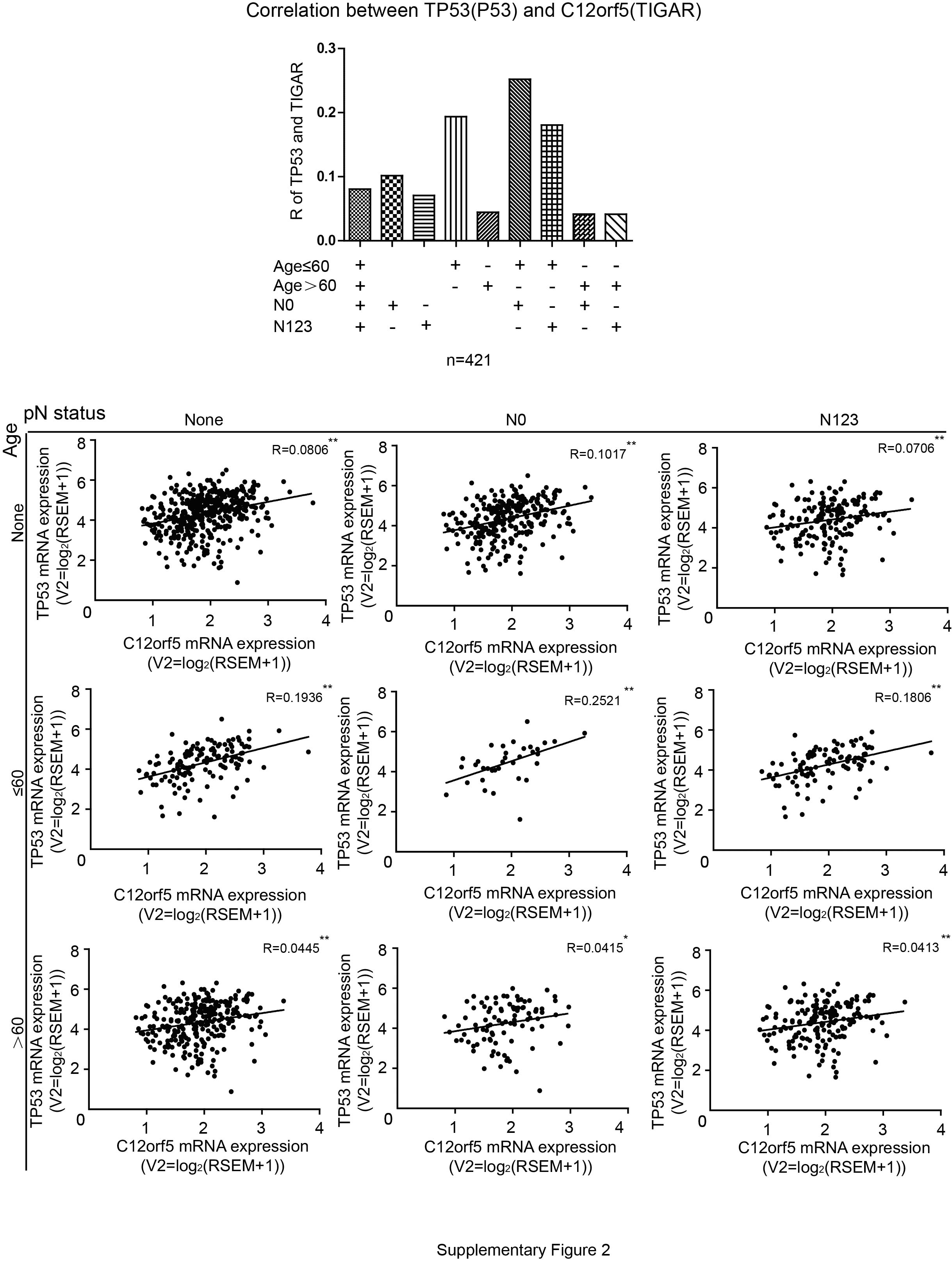

Supplement: Figure S2 — Correlation of TP53 (P53) and C12orf5 (TIGAR) expressions. Correlation of mRNA expression between TP53 (P53) and C12orf5 (TIGAR) was analyzed using sequencing data retrieved from TCGA (The Cancer Genome Atlas) STAD (stomach adenocarcinoma) dataset in UCSC Xena repository. It appeared that correlation of TP53 and C12orf5 expression turned out to be more significant after filtering by age (≤ 60) and N (N0). [file Image_2.JPEG]
